# Consequences of Coronavirus pandemic on the image of nursing in Iran

**DOI:** 10.1002/nop2.890

**Published:** 2021-05-03

**Authors:** Mojgan Lotfi, Vahid Zamanzadeh, Afsaneh Nobakht, Mohammad Khajehgoodari

**Affiliations:** ^1^ Department of Medical Surgical Nursing Faculty of Nursing and Midwifery Sina Hospital Tabriz University of Medical Sciences Tabriz Iran; ^2^ Department of Medical Surgical Nursing Faculty of Nursing and Midwifery Tabriz University of Medical Sciences Tabriz Iran; ^3^ Faculty of Nursing and Midwifery Sina Hospital Tabriz University of Medical Sciences Tabriz Iran; ^4^ Tabriz Valiasr International Hospital Tabriz University of Medical Sciences Tabriz Iran

The image of nursing is closely related to the identity and role of the nurse, cultural background, clinical practice, job satisfaction and quality of care. One of the long‐term challenges of the nursing profession is the general public image of this profession. The image is the assumption and perception that the general public has about a person and a profession or organization (Farsi et al., 2010). A positive public image has a great effect on attracting social and environmental support. Negative public stereotypes of nursing lead to feelings of frustration and confusion about self‐image and social identity and can create a repressive environment for the profession (Kaur Pushpinder & Rawat, 2017).

In Iran, more than 100 years have passed since the academic training of nursing. In 1916, the first nursing school in Iran was established in Tabriz, which remained the only nursing programme in Iran until 1935, after which the government established four nursing schools in major cities. During the years of the Iran–Iraq war (1980–1988), the need to increase the number of nursing schools and the recruitment of men in this field was felt. Also, master's and PhD programmes in nursing under the supervision of the Nursing Board have grown significantly in recent decades (Khomeiran & Deans, 2007; Nasrabadi & Emami, 2006; Nasrabadi et al., 2004). However, the professional growth of nursing in Iran until recently has not been able to significantly improve the image of nursing (Cabaniss, 2011; Heilemann, 2012).

Since the start of the Corona pandemic in 2020, nurses have been in a global arena to fight this deadly disease, an event that can be described as one of the most important crises in human history. What is often overlooked, and what the pandemic of COVID‐19 disease reminds us of, is the perpetual role of nurses in the healthcare system. The process has accelerated with the outbreak of this disease. Also, in a year when the important role of nurses has received more attention (CDC, 2020), the World Health Organization (WHO) has named 2020 the "Year of Nurses and Midwives." (World Health Day, 2020). The World Bank blog group also wrote on its front page: “Nursing is very important for COVID‐19 and global health,” and the image of injuries and wounds of nurses was exposed to everyone who suffered due to long shifts in the care of patients with COVID‐19. Finally, many artistic images of nurses' courage and self‐sacrifice were shared on social media, making them a myth in the war against Corona (Speaker & Moffatt, 2020).

In our country, various groups are active in the fight against the widespread COVID‐19, and the front line of this fight is in the hands of the medical community. Nurses, as an important part of this community during the COVID‐19, recognized their true value and role to patients, colleagues and the community. In addition to the manifestation of human traits, especially in nurses who sacrificed their lives, dignity and family issues with self‐sacrifice, love and interest in work and hospital, COVID‐19 was a good opportunity to show the capacity, ability and position of individuals and to reveal many hidden capacities (Sadati et al., 2020).

If we skip the bitter and deadly events of COVID‐19 and see its positive points in a separate window, we encounter several positive points. One of these positive points is showing the position of the health team in the community and improving the social status of nurses such as a more accurate presentation of the nursing profession to the community, the importance of paying attention to increasing wages and benefits, nurses 'participation in policy and political affairs, increasing the number of nurses, improving nurses' working conditions and creating a participatory environment.

This is a vital period in the history of nursing where nurses can maintain and develop this image and force the government to correct the decisions made about them. However, huge actions and activities are still needed to consolidate achievements and solve social, cultural and economic problems. Because nurses in this unequal war are in the most challenging economic conditions and insecure conditions despite this favourable aspect.

## CONFLICT OF INTEREST

The authors declare that they have no conflicts of interest.

## RESEARCH ETHICS COMMITTEE APPROVAL

None.

## Data Availability

All information in this letter to the editor can be made available to all readers.
